# Diagnostic Challenges and Management of Blunt Traumatic Duodenal Diverticulum Perforation: A Systematic Review

**DOI:** 10.3390/jcm15114390

**Published:** 2026-06-05

**Authors:** Maciej Rybicki, Bartłomiej Białas, Karol Kamil Kłosiński, Zbigniew Włodzimierz Pasieka, Bartosz Marek Czyżewski, Piotr Tomasz Arkuszewski

**Affiliations:** 1 Students’ Research Group, Department of Biomedicine and Experimental Surgery, Faculty of Medicine, Medical University of Lodz, Narutowicza 60, 90-136 Lodz, Poland; maciej.rybicki@stud.umed.lodz.pl (M.R.); bartlomiej.bialas@stud.umed.lodz.pl (B.B.); bartosz.czyzewski@student.umed.lodz.pl (B.M.C.); 2 Plastic, Reconstructive and Aesthetic Surgery Clinic, Institute of Surgery, Medical University of Lodz, 90-419 Lodz, Poland; 3 Department of Biomedicine and Experimental Surgery, Faculty of Medicine, Medical University of Lodz, Narutowicza 60, 90-136 Lodz, Poland; karol.klosinski@umed.lodz.pl (K.K.K.); zbigniew.pasieka@umed.lodz.pl (Z.W.P.)

**Keywords:** blunt abdominal trauma, duodenal diverticulum perforation, gastrointestinal surgery, computed tomography, systematic review

## Abstract

**Background/Objectives**: Traumatic duodenal diverticulum perforation is a rare, potentially fatal consequence of blunt trauma. Nonspecific symptoms and diagnostic challenges often delay recognition. This systematic review characterizes its clinical features, management, and outcomes. **Methods**: A systematic literature search covering 1960 to December 2025 identified eligible cases. Inclusion criteria were blunt trauma-related duodenal diverticulum perforations confirmed by imaging or surgery. Data were analyzed according to PRISMA 2020 guidelines. **Results**: Twenty-one cases were identified (mean age 62.9 years; sex ratio (M:F) 8:13). Primary injury mechanisms were traffic accidents (10 of 21) and falls from height (8 of 21). Most injuries involved the descending duodenum (D2; 17 of 21). Common presenting signs included abdominal pain (19 of 21) and epigastric tenderness (16 of 21). Computed tomography confirmed findings consistent with perforation in all scanned patients (17 of 17). Surgical management was employed in 20 of 21 patients, predominantly via manual (11 of 20) or stapled (9 of 20) diverticulectomy, with drainage applied in 18 of 20 operated cases. Complications occurred in 13 of 21 patients. Overall mortality was 4 of 21. **Conclusions**: Traumatic duodenal diverticulum perforation remains a life-threatening event requiring high clinical vigilance. The data collected suggest that early CT scanning and prompt surgical intervention may be associated with better treatment outcomes, although these conclusions should be treated with caution due to the small sample size. The protocol was registered in PROSPERO (CRD420261285658).

## 1. Introduction

Traumatic injuries (blunt and penetrating) and their complications are the fourth leading cause of death worldwide and the leading cause of death in the first half of life. Between 9% and 17.75% of all injuries are abdominal injuries [1,2]. The liver and spleen are the most often injured, accounting for 50 to 60% of abdominal trauma, and most of these result from blunt trauma [3]. Up to 10% of all trauma cases reported to emergency departments are blunt abdominal injuries. Most of them are victims of traffic accidents, which are common, and knowledge of their pathophysiology is crucial in clinical practice [3].

Much rarer, but no less important, abdominal injuries include injuries to the bladder (2.3%), pancreas (1.3%), adrenal glands (0.4%), stomach (0.2%), and diverticula, including duodenal diverticula [1].

### 1.1. Characteristics of Duodenal Diverticula

A diverticulum is a bulge in the wall of the intestine. The most well-known are duodenal diverticula, Meckel’s diverticula and colonic diverticula. However, diverticula can occur anywhere in the digestive tract, even in the caecum [4,5,6,7].

Diverticula most commonly affect the large intestine, but it should be noted that small intestine diverticula are probably underestimated, as their location often remains invisible in imaging studies. Small bowel diverticula are most commonly located in its anterior section—the duodenum. They are found in 7–23% of patients undergoing endoscopic retrograde cholangiopancreatography (ERCP) and in 20–22% of patients in autopsy studies, making them the second most common location of diverticula in the gastrointestinal tract [8,9,10,11,12,13]. They were first described in 1710 by Chomel [14].

In order to accurately characterize duodenal diverticula, it is necessary to understand its anatomical structure. The duodenum consists of four parts:

- Ampulla (D1);
- Descending part (D2);
- Horizontal part (D3);
- Ascending part (D4).

Importantly, the first 2–3 cm (proximal part of D1) are located intraperitoneally, while the remaining part of the duodenum (distal D1 and D2–D4) is retroperitoneal. This location and proximity to other organs means that both primary and secondary processes can occur within the duodenum [15]. Duodenal diverticula are most commonly found approximately 2 cm from the papilla of Vater (D2)—in 62% of cases—which is explained by the structural weakness of the wall in this area [16]. The second most common location is the D3 segment (30%), approximately 8% of diverticula are located in D4, and less than 1% in the duodenal ampulla (D1) [13].

The vast majority (90–95%) of duodenal diverticula are asymptomatic and are detected incidentally in imaging studies [17,18]. There are various classifications of duodenal diverticula, but the most common is based on origin: congenital and acquired. Congenital (true) diverticula develop during the prenatal period and have a wall consisting of four layers (mucosa, submucosa, muscularis, and serosa), while acquired (false) diverticula, which develop after the second decade of life, have only mucosa and serosa. Congenital diverticula account for approximately 10% of all cases, while acquired diverticula account for as much as 90% [13].

Perforations of duodenal diverticula are extremely rare—to date, approximately 200 cases have been reported [19]. The most common cause of perforation is diverticulitis or ischemia caused by food retention. Ulceration, iatrogenic injury, the presence of a foreign body, or other traumatic factors are observed much less frequently [20]. Traumatic causes are among the rarest and occur with a frequency comparable to that of injuries to the entire duodenum, amounting to 3–5% [21]. Despite the small number of cases, traumatic perforation of a duodenal diverticulum has serious clinical and diagnostic consequences. The symptoms are nonspecific—abdominal pain, vomiting, and fever—and may mimic other, much more common conditions such as acute cholecystitis, pancreatitis, appendicitis, or peptic ulcer disease [9,16,22]. It should be emphasized that overlooking such cases may lead to delayed diagnosis and the development of severe complications, including patient death. The most common mechanism of injury leading to duodenal diverticulum perforation is direct impact to the epigastrium, typical of traffic accidents, falls from height, or blunt trauma caused by impact.

### 1.2. Research Gap and Rationale for Systematic Review

The available data on duodenal diverticular perforation resulting from blunt trauma are extremely limited and scattered. To date, only 21 cases have been described in the professional literature, mainly in the form of individual reports or short case series [23,24,25,26,27,28,29,30,31,32,33,34,35,36,37,38,39,40,41,42,43]. It is worth noting that the vast majority of the cases described come from recent years, which may be related to both increased detectability thanks to better diagnostic methods and increased interest in this rare pathology in the clinical setting.

Previous review papers present only tabular summaries, without a full comparative analysis or comprehensive synthesis of data [41,42]. The lack of a systematic study prevents a reliable assessment of clinical features, treatment methods, and patient survival rates. No uniform diagnostic criteria or descriptions of clinical symptoms characteristic of this disease entity have been developed.

Furthermore, no comparison has been made between cases of congenital and acquired diverticula, which limits our understanding of the differences in clinical course and prognosis. Insufficient knowledge of this rare pathology may lead to delayed diagnosis and severe clinical consequences, including patient death.

This paper focuses exclusively on blunt traumatic injuries, as iatrogenic injuries are usually easier to predict—their mechanism and location are known at the time of medical intervention—while penetrating trauma allows for direct determination of the site of injury and suspicion of the cause. In the case of blunt injuries, however, the mechanism of force is often complex and difficult to determine unequivocally, making the diagnosis of duodenal diverticular perforation a significant diagnostic challenge. Therefore, a systematic review of the literature covering all published cases to date is both scientifically and clinically justified.

Updating and standardizing the data will allow for a better understanding of the clinical course, identification of symptoms and prognostic factors, and increased awareness among clinicians regarding this rare but potentially fatal disease entity.

### 1.3. Objectives and Methodology

The objective of this systematic review, developed in accordance with the Preferred Reporting Items for Systematic Reviews and Meta-Analyses (PRISMA) 2020 guidelines (detailed in Supplementary Table S1), was to collect, analyze, and synthesize all published cases of duodenal diverticulum perforation resulting from blunt trauma [44]. The protocol for this review has been registered in the International Prospective Systematic Reviews Register (PROSPERO) and assigned the number CRD420261285658. The analysis included 21 cases, focusing on the clinical characteristics of this rare condition by collecting data on patient demography, mechanism of injury, clinical symptoms, diagnostic and treatment methods, and patient outcomes. Ultimately, the synthesis of these data allowed for the creation of a comprehensive clinical picture to increase awareness among clinicians regarding the diagnosis and management of patients with this rare but potentially dangerous condition, which should not be underestimated.

## 2. Methods

This study did not require the approval of a bioethics committee due to its retrospective nature.

### 2.1. Eligibility Criteria

The review included exclusively retrospective papers describing case reports and case series concerning duodenal diverticulum rupture as a result of blunt trauma, such as traffic accidents or falls from height. Stab wounds, iatrogenic injuries, and injuries of unclear etiology were excluded because in these cases the location and depth of the injury are usually known, allowing for rapid diagnosis and surgical intervention. In the case of blunt trauma, diagnosis is more difficult because the injuries are hidden and clinical decisions require observation and careful interpretation of symptoms, which increases the importance of a retrospective review of the relevant literature.

Only publications in which duodenal diverticulum rupture had been confirmed surgically or by using imaging tests were included. No age, gender, or ethnic restrictions were applied. The time criteria included publications issued between 1960 and 2025, in all languages. Only articles with an available abstract were included; the full texts of the publications were manually reviewed by two independent reviewers, who decided whether to include or exclude the studies.

Data from published studies were clustered for analysis according to the location and magnitude of diverticula, the presence of clinical symptoms (with analysis of frequency and specificity), the surgical treatment used, and patient survival. In cases where data were missing, the information was noted, but the cases were not excluded due to the rarity of the observations.

### 2.2. Information Sources

The literature search was conducted in the following databases: PubMed/MEDLINE, Embase (OVID), Scopus, Web of Science, and Cochrane Library. Additionally, a supplementary search was performed in Google Scholar. Where possible, the OVID interface was used. The time range comprised publications from 1960 to 2025.

In addition, reference lists (bibliographies) of included articles were reviewed to identify potential additional cases. No contact with authors was planned to supplement missing data; all information was obtained directly from publications.

### 2.3. Search Strategy

The search strategy included combinations of keywords and MESH terms, including:

- “duodenal diverticulum” OR “duodenal diverticula,”
- AND (*trauma* OR *injury* OR *traumatic* OR *blunt trauma* OR *perforation* OR *perforated* OR *crush OR compression OR rupture OR laceration*).

Various grammatical forms, plural forms, and synonyms were included in order to capture as many relevant publications as possible. The strategy was designed to capture all publications that met the inclusion criteria. Its effectiveness was preliminarily verified based on a set of reference articles. The detailed search strategies for all databases are provided in Supplementary File S2.

### 2.4. Selection Process

Each record (title/abstract) was evaluated by two independent reviewers. In case of disagreement, the decision was resolved through discussion; if no consensus was reached, a third independent reviewer made the final decision.

**Figure 1:**
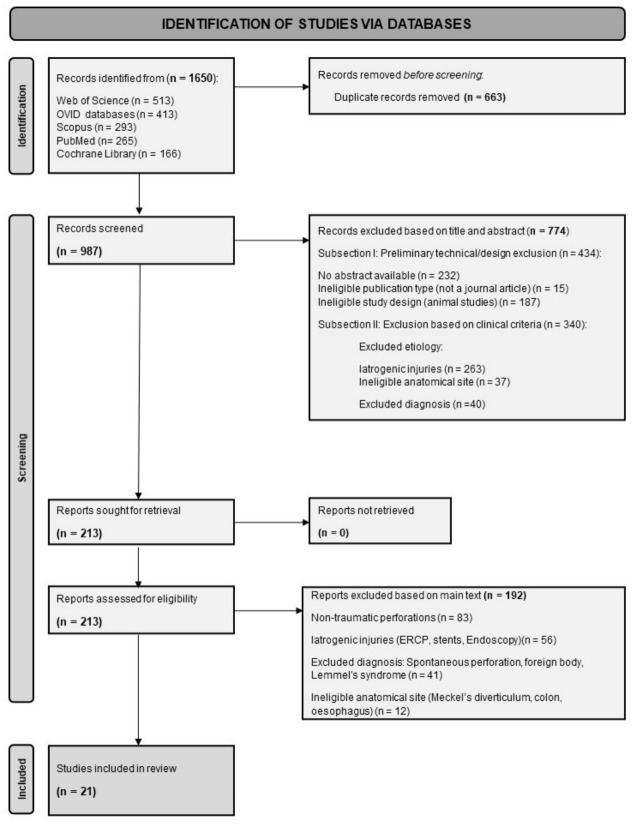
PRISMA 2020 flow diagram outlining the systematic review selection process.

The search was not restricted to articles in English. Papers in non-Polish and non-English languages were translated using automated tools and the translations were verified for terminological accuracy by experts in the medical fields. Duplicate data were excluded: if the same case was described in more than one publication, only the original data from the author describing the case were included. The selection process covered the period from 1 September 2025 to 31 December 2025. The article selection process is presented in the PRISMA diagram in Figure 1.

### 2.5. Data Collection Process

The data were collected independently by two independent reviewers, and any discrepancies were resolved by a third reviewer. The following variables were collated:

- Demographic: age, gender;
- Traumatic: cause of injury, mechanism (e.g., compression, deceleration, shearing injuries);
- Clinical and diagnostic: location of the diverticulum, size, nature (congenital/acquired), clinical symptoms (abdominal pain, vomiting, epigastric tenderness, peritoneal symptoms, elevated temperature, others), diagnostic method, histopathological examination result (if performed);
- Therapeutic: surgical treatment method, presence of drainage, complications, complications (retrospectively graded by the authors using the Clavien–Dindo classification system);
- Course: length of hospitalization, patient outcome (survived/died).

The data were compiled in Excel tables and analyzed using Excel and Statistica. EndNote was used for source management. In case of conflicts between reports, the hierarchy of primary data from the author was adopted. Missing data were noted, but cases were not excluded in order to preserve the full value of rare observations.

### 2.6. Bias Risk Assessment

Bias risk assessment was performed for all included publications using the Joanna Briggs Institute (JBI) Critical Appraisal Checklist for Case Reports/Case Series.

Two independent reviewers performed the assessment; any disagreements were resolved by a third reviewer. No tools were used to automate the assessment of bias. The results are presented in a table including: year of publication, type of study, tool used, and assessment result.

### 2.7. Measures of Effect

For binary data, such as patient survival or the occurrence of complications, proportions and frequencies were used. For continuous variables, such as length of hospital stay or diverticulum size, medians, modal values, means, and standard deviations were analyzed. The results are presented in absolute numbers.

### 2.8. Data Synthesis Methods

The data are presented in tables, grouped according to: location of diverticula, size, presence of symptoms, and treatment used. The analysis included:

- A summary of symptoms, characteristics of diverticula, treatment methods, and treatment outcomes (survival/complications);
- Analysis of the frequency and specificity of clinical symptoms;
- Evaluation of complication severity based on the assigned Clavien–Dindo grades in relation to time-to-intervention;
- Narrative synthesis of data to identify the most common and rare symptoms.

Due to the small number of cases (n = 21), no meta-analysis or formal analysis of heterogeneity or subgroups was performed.

### 2.9. Assessment of Publication Bias and Certainty of Evidence

Systematic assessments of risk of bias were conducted using the Joanna Briggs Institute (JBI) Critical Appraisal Checklist for Case Reports. Given the retrospective nature of the analyses and the small number of available cases, no formal assessment of publication bias or certainty of evidence (e.g., using the GRADE approach) was performed.

## 3. Results

### 3.1. Risk of Bias Results

Systematic bias risk assessments were performed using the Joanna Briggs Institute (JBI) Critical Appraisal Checklist for Case Reports, which is appropriate for case report studies. The analysis covered 21 publications describing cases of duodenal diverticulum perforation after blunt abdominal trauma. The assessments were performed independently by two researchers, and any discrepancies were resolved by consensus.

Based on the JBI checklist, all the studies assessed scored highly, indicating the high quality of the case reports presented. It should be emphasized that despite meeting these reporting criteria, case reports are, by their very nature, low-level evidence and remain highly susceptible to inherent biases associated with publication and sample selection. Fourteen of the twenty-one studies received the maximum score in the assessment, while the average total consensus score on the JBI scale was 15.1 points (range: 12–16).

The most frequently met JBI criteria included:

- Complete description of patient demographics;
- Detailed description of the intervention used;
- Presentation of adverse events described and a clear formulation of clinical conclusions (take-away lessons).

The most common incomplete or omitted elements concerned: the description of the patient’s condition after the intervention (post-intervention condition) and the presentation of diagnostic test results. Furthermore, none of the studies included in the analysis received external funding.

The agreement between assessors was very high, with an average agreement coefficient of 0.98, confirming the consistency and reliability of the systematic error risk assessment.

The detailed results are also presented in tabular form, including subcategories and the opinions of two independent reviewers, in Table 1.

### 3.2. Study and Population Characteristics

The literature search has identified 21 cases of duodenal diverticulum perforation following blunt trauma, all described in publications meeting the PRISMA inclusion criteria [44]. All studies were case reports or short series.

The mean age of patients was 62.9 (SD 14.3) years (range 33–84; median 64; and modal 64). There were 13 women and 8 men, corresponding to a F:M ratio of 1.6:1.

The most common mechanism of injury was a traffic accident (n = 10), followed by a fall from height (n = 8) and direct blunt abdominal trauma (n = 2). In one case, perforation occurred as a result of pressure on the abdomen.

The location of the diverticulum was most often in the descending part of the duodenum (D2) (n = 17), less often at the junction of the D2 and D3 (n = 2) and fourth (D4) parts (n = 1). Most diverticula were acquired (pseudodiverticula), while individual descriptions concerned true diverticula.

### 3.3. Clinical Symptoms and Course

Abdominal pain was the most common symptom (n = 19) [23,24,25,26,27,28,29,30,31,32,33,35,37,38,39,40,41,42,43], followed by epigastric tenderness (n = 16) [23,24,25,26,27,28,29,30,31,33,35,37,39,40,41,42]. Peritoneal symptoms occurred in ten cases [23,24,27,28,30,31,35,40,41,42], and vomiting or nausea in five patients [27,29,31,35,41].

The time of hospitalization was 17.3 (SD 16.8) days on average (range 3–68; median 11.5), suggesting delayed onset of symptoms or slow progression in some patients. In many cases, patients were admitted with symptoms of peritonitis. Detailed demographic data, injury mechanisms, and clinical presentation are summarized in Table 2 and fully detailed for each individual patient in Table 3.

The vast majority of patients in this group underwent surgical treatment within the early intervention window (<24 h from injury), with procedures frequently reported as emergency operations or performed within a few hours of the patient’s arrival. On the other hand, in a small group of patients, there was a delay in diagnosis and treatment (>24 h), which was often due to late presentation at hospital or initial conservative management. Postoperative complications, assessed retrospectively according to the Clavien–Dindo classification, varied considerably. Whilst nearly half of the patients (10/21) recovered without complications, eight patients experienced serious complications (grade ≥ IIIa), including four deaths (grade V).

A crucial aspect of the analyzed data is the presence of concomitant injuries, which were recorded in 7 out of 21 patients; three of these cases were classified as severe multiple organ trauma involving simultaneous injuries to the chest, abdomen, cervical spine, or orthopedic injuries. It should be emphasized that the presence of concomitant injuries, and in particular severe multiple organ injury, constitutes an independent prognostic factor with a significant impact on overall morbidity and mortality. Importantly, half of the fatal cases (2/4), as well as several cases of severe, life-threatening complications (e.g., grade IVa), occurred in patients with concomitant injuries. This highlights that clinical outcomes and prolonged hospital stays are often determined not only by the diverticular perforation itself, but by the cumulative physiological burden resulting from multiple organ injuries. The clinical management, time to intervention and outcomes are presented in detail in Table 4.

### 3.4. Treatment

Surgical treatment was used in 20 out of 21 patients. Only one of the patients was treated conservatively [38].

The most common surgical treatment was manual diverticulectomy (n = 11) [23,24,25,26,27,30,32,33,34,35,37]. In nine cases, diverticulectomy was performed using staplers [28,29,31,33,36,39,41,42,43]. Drainage was used in eighteen patients who underwent surgery. It is noteworthy that only two patients (2/20) who underwent surgery did not require drainage [33,39].

The analyzed reports describe the use of three main imaging methods in the diagnosis of duodenal diverticular perforation after blunt trauma: computed tomography (CT), ultrasound and X-ray.

CT was the most commonly used method—in seventeen cases, it confirmed perforation. In all cases described in which CT was performed, the examination provided direct or indirect imaging features consistent with perforation (e.g., the presence of free air and/or leakage of intestinal contents).

Ultrasonography was used in five cases [24,28,34,37,38]. In three of these five examinations (3/5), features suggestive of perforation were found [28,37,38]. In contrast, no signs of perforation were found in two ultrasound examinations [24,34]. Abdominal X-ray examination was performed in five cases [24,26,30,35,37]. Only two of the five examinations showed signs of perforation on X-ray (free air) [26,30].

### 3.5. Treatment Outcomes and Complications

The average length of hospitalization was 17.3 ± 16.8 days (median 11.5 days), ranging from 3 to 68 days. Complications occurred in a total of thirteen patients. Minor complications, including postoperative wound infection and periduodenal abscess, were reported in five patients [23,24,29,30,33], while severe complications, such as biliary fistula, sepsis and multiple organ failure, occurred in eight patients [25,26,27,32,35,36,40,43].

However, there were no complications in the remaining eight cases [28,31,34,37,38,39,41,42].

The total mortality rate was four patients [25,26,27,32], all of them during postoperative treatment. In the analyzed cases, the risk factors for death included advanced age, location of the diverticulum in the descending duodenum (D2), presence of sepsis and delayed diagnosis. Full recovery was reported in seventeen patients.

Histopathological data were available in ten cases [25,27,28,29,35,36,37,39,41,42], while in eleven cases they remained incomplete. Among the available histopathological results, three congenital diverticula [25,27,42], and seven acquired diverticula were identified [28,29,35,36,37,39,41]. The follow-up period after treatment ranged from one to twelve months (mean 5.4 months), with follow-up data being incomplete.

Comprehensive data regarding diagnostic modalities, surgical management, and clinical outcomes are presented in Table 5.

### 3.6. Study Limitations

This systematic review has several important limitations that should be considered when interpreting the results.

Firstly, the data contained in the case reports are heterogeneous, and some important variables (e.g., the exact time from injury to intervention, the extent of perforation, associated injuries (polytrauma), ICU admission, cause-specific mortality, imaging diagnosis or detailed histopathological results) were not reported consistently. As a result, not all parameters could be included in the statistical analysis.

Secondly, the analysis is based solely on individual case reports and short series, which limits the generalizability of the results. Such publications often present exceptional cases, which introduces reporting bias, as more severe or unusual cases are more likely to be reported in the literature.

Thirdly, the small number of cases (n = 21) makes it impossible to perform a reliable comparative analysis between groups (e.g., surgical vs. conservative treatment) or to calculate the statistical significance of differences (χ^2^, Fisher’s, Mann–Whitney tests). The lack of raw data in most publications also makes it impossible to perform a formal quantitative meta-analysis, calculate the cumulative effect or use a random effects model.

Fourthly, differences in the quality of data reporting (e.g., lack of follow-up information in some cases) and population heterogeneity (age, mechanism of injury, location of diverticulum) mean that the analyzed data should be treated as descriptive rather than comparative.

Despite these limitations, the review provides a valuable, synthetic summary of cases of duodenal diverticulum perforation following blunt trauma, which, due to the rarity of the problem, is currently the most up-to-date source of clinical knowledge in this field.

## 4. Discussion

### 4.1. Presentation of Results in the Context of Literature

The following systematic review fills an important research gap. Until now, there has been no comprehensive clinical and statistical synthesis of cases of post-traumatic duodenal diverticulum perforation. The professional literature is dominated by individual case reports or small series (e.g., reviews of perforated duodenal diverticula). From this perspective, our work represents the first systematic collection of data allowing for a comprehensive overview.

First of all, we would like to point out that by using the term ‘trauma’ we refer to an external factor causing damage (e.g., a traffic accident, abdominal crushing, a fall from a height), while ‘injury’ means specific damage to the body resulting from trauma (e.g., a wound, fracture, perforation).

The notable predominance of locations in the D2 segment (17/21) deserves discussion. We hypothesize that such results are supported by the anatomical structure: part D2 of the duodenum lies retroperitoneally, in close proximity to vascular and biliary structures, which may hinder gas flow, promote local infection and thus increase susceptibility to perforation. The literature emphasizes that duodenal diverticulum perforation most often affects parts D2 and D3, which may be consistent with the above hypothesis [45].

Furthermore, in the cases we analyzed, the clinical presentation could be misleading and resemble other diseases (e.g., suspected peptic ulcer disease—Brabrand et al.) [35]. This may lead to delayed diagnostic and therapeutic decisions. The literature also highlights that the diagnosis of perforated duodenal diverticula is difficult, particularly due to their retroperitoneal location and non-specific symptoms [46].

Due to the fact that the duodenum is an organ partially situated in the retroperitoneal space, early leakage of intestinal contents following rupture is initially confined to this space. Consequently, classic peritoneal symptoms may appear with a delay and may be completely absent during the initial assessment [41,42,47]. In the group of patients we analyzed, markedly delayed peritoneal symptoms were noted in three cases, appearing from 4 h to as long as 2 days after the injury. These delayed clinical symptoms directly hinder diagnosis and increase the risk of delayed therapeutic intervention [48]. Therefore, clinicians need to remain highly vigilant, as the absence of abdominal rigidity immediately following blunt trauma does not rule out duodenal diverticular perforation with absolute certainty [41,42,48].

Demographically, in the majority of cases, injuries affect men, which may be linked to a greater propensity to take risks and greater communication and professional activity exposed to injury [49,50,51]. Regarding sex reporting, both male and female cases were included in this analysis. Demographically, blunt abdominal trauma predominantly affects males due to higher occupational and risk-taking exposures. However, our cohort demonstrated an unexpected female predominance (M:F ratio 8:13). Given the highly limited sample size (n = 21), this disproportion is most likely a statistical artifact rather than a true reflection of sex-based anatomical or physiological predispositions to duodenal diverticulum perforation.

The mechanisms by which blunt abdominal trauma can lead to injury of an intestinal diverticulum involve several distinct, though often coexisting, pathophysiological processes.

The first hypothetical mechanism that may contribute to post-traumatic perforations of the duodenal diverticulum is abdominal compression. Blunt trauma can damage the diverticulum by direct compression of the intestine against the spine and secondary increase in pressure within the intestinal lumen. This mechanism, also known as the closed loop phenomenon, has been identified as the direct cause of perforation of other various intestinal diverticula following blunt trauma [7,19,52]. Furthermore, during blunt abdominal trauma, compression generates stretching (tensile) forces perpendicular to the compressive forces [53]. In such a circumstances—with evident pressure on the abdominal cavity—Meckel’s diverticula ruptured following blunt trauma: impact with the bicycle handlebars [54,55,56,57], punch and kick in the abdomen [58,59,60,61], stopping by seat belts [52,62], being struck in the abdomen by a piece of wood while cutting [63], crushed against the wall by a lorry [64], and a blow to the abdomen from the plow handle [65].

Another pathophysiological mechanism potentially responsible for injury is sudden deceleration. It is typical for traffic accidents and falls from height to generate shearing forces between the mesentery and the intestinal wall. Under these circumstances, sudden deceleration or acceleration causes the intestine to continue moving relative to its fixed points [52,55,62]. Intra-abdominal adhesions increase the risk of injury to the intestine and mesentery following blunt abdominal trauma [66]. Theoretically, during deceleration, it is possible to pull the diverticulum through adhesions in the duodenal area, or the violently moving duodenum will be held in place by tensioned adhesions, which results in diverticulum rupture. Duodenal adhesions have been described in the literature [67]. It is also worth noting another potential mechanism of duodenal diverticulum rupture resulting from deceleration associated with the sudden displacement of abdominal organs. In such cases, mechanical rupture of duodenal diverticulum can be caused by one of the moving adjacent structures. This would be even more likely if the diverticulum was affected by a chronic inflammatory process. There are known cases where a duodenal diverticulum has compressed the bile ducts [68].

In some cases, the mechanism of injury strongly suggested direct abdominal compression: a kick to the abdomen [28,39] or impact of the abdomen against a balcony railing [42]. In other cases, descriptions of the details of the trauma suggest a compression mechanism during car accidents, hypothesizing an impact with the passenger seat in front/steering wheel or being held down by a seatbelt [25,26,29,33,34,36,37,38,41]. Conversely, among traffic accidents, only the case described by Caires et al. referred to the circumstances of the injury in greater detail, pointing to the direct impact of pressure from seat belts [33]. Nevertheless, it should be pointed out that deceleration could have been dominant in such cases. Likewise, in falls from height, it cannot be ruled out that deceleration was accompanied by compression [23,24,27,30,31,32,35,40].

The correlation between the location of the diverticulum (most commonly D2) and the risk of rupture may also be related to anatomy: the D2 segment is retroperitoneal, less mobile and may be more susceptible to compressive or shearing forces. Additionally, in the literature, duodenal diverticula are most often classified as acquired diverticula (false diverticula)—which have a thinner wall than congenital diverticula (true diverticula) and may therefore be less resistant to internal or external forces acting on the intestine [69]. However, our material lacks adequate histopathological data to fully distinguish between the types of diverticulum and rupture and to apply this classification in clinical analysis.

**Figure 2:**
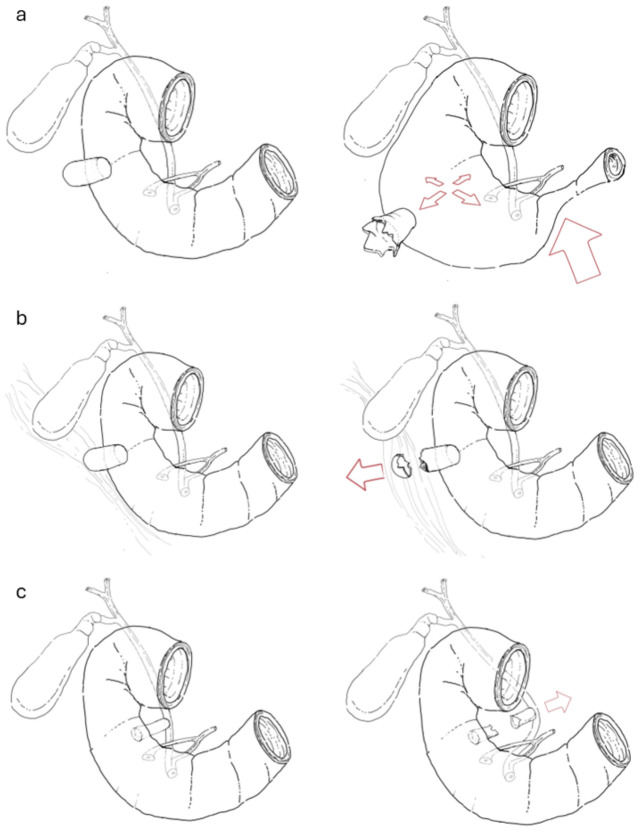
Duodenal diverticular perforation: proposed mechanisms and anatomical considerations. (**a**) **Typical anatomy of a duodenal diverticulum situated in segment D2, demonstrating its relationship with the biliary tree.** Compression-related injury: blunt abdominal trauma leads to a sudden increase in pressure within the duodenal lumen (the so-called closed-loop phenomenon). This can result in rupture of the diverticulum wall. The red arrows indicate the vectors of the pressure forces acting on the diverticulum. (**b**) **Duodenal diverticulum with peri diverticular adhesions, possibly related to chronic inflammation or a history of diverticulitis.** Hypothesized rupture of the diverticulum induced by traction transmitted through adhesions, resulting from tensile and shear forces acting on relatively stationary, retroperitoneal segments of the duodenum during sudden braking. The arrow indicates the direction of the traction forces. (**c**) **Typical anatomical position, in close proximity to the bile ducts.** Potential mechanical perforation of the diverticulum caused by traction from the bile ducts, where displacement or sudden pressure increase within the biliary system can contribute to rupture of the diverticulum wall. The arrow indicates the direction of the traction forces. Notably, in our limited data, computed tomography (CT) showed consistent detection of perforation features in radiological images where utilized, which temporally coincides with zero mortality over the last 12 years in the reported cases. However, it should be emphasized that advances in medicine, improved diagnostic and therapeutic procedures, and access to other advanced imaging methods have also contributed to the improvement in outcomes, which should be taken into account in the interpretation.

To better demonstrate the suggested mechanisms by which blunt trauma can lead to perforation of the duodenal diverticulum, a schematical overview is presented in Figure 2.

Moreover, it is worth noting that the cases analyzed span the period from 1960 to 2025, which significantly affects both therapeutic options and diagnostics. Nowadays, imaging tests such as (Extended) Focused Assessment with Sonography in Trauma (FAST or eFAST) or TraumaScan (CT) are standard, and in the vast majority of cases they allow for early diagnosis and treatment. However, some of our cases date back to a time when these methods were not in widespread use, which requires caution when comparing results and assessing diagnostic progress.

In short: our analysis suggests that post-traumatic duodenal diverticulum perforation, although rare, may present with a characteristic profile—D2 location predominance, difficult diagnosis, delays in treatment and the need for rapid intervention. The results are consistent with the literature on perforated duodenal diverticula, although there is very little data available on blunt trauma alone, which makes our synthesis valuable and unique.

### 4.2. Limits of the Evidence Contained in the Review

One of the main limitations is the considerable heterogeneity of the data—the patients described in individual publications came from different regions of the world and were described by different teams over a period spanning more than six decades (1960–2025). This introduces substantial temporal heterogeneity, particularly regarding the evolution of diagnostic imaging (e.g., the advent of modern CT), trauma management protocols, and surgical techniques. In many cases, key information regarding diagnosis, treatment, complications and symptomatology was missing. Furthermore, a significant number of articles did not take into account comorbidities that were likely to have affected the patient’s condition and the course of the disease, making it difficult to assess mortality risk and other clinical outcomes.

Another relevant issue is the lack of data on the structure of perforated diverticula. Histopathological diagnosis was available in only 10 out of 21 cases. The lack of comprehensive reporting of histopathological results limits the ability to distinguish between types of diverticula (congenital vs. acquired) and their relationship to clinical course.

It should be emphasized that post-traumatic ruptured duodenal diverticula are an extremely rare and atypical phenomenon. Therefore, the literature predominantly reports cases with a more severe or atypical course, while groups of patients with a milder course may be underrepresented. This selection may lead to publication bias and an overestimated picture of clinical severity.

Another limitation is the fact that in some cases the injuries (e.g., traffic accidents, falls from height) were very extensive and affected many areas of the body, which in itself could have substantially prolonged hospitalization and/or indirectly contributed to the patient’s death. In the analysis, we must take into account that the diverticular rupture itself may have been only one of many elements of severe injury (e.g., the case of Kim et al.—acute subdural hemorrhage, liver rupture, renal failure, sepsis, multiple organ failure resulting in death) [32]. This type of polytrauma makes it difficult to isolate the impact of the diverticulum as a single entity.

Moreover, data on the time from trauma to intervention may in some cases have been estimated rather than accurately measured, which limits their reliability and prevents them from being treated as the main subject of analysis. Variables dependent on geographical factors, medical developments in a given region and the quality of healthcare were also not taken into account, as these factors are unmeasurable or difficult to estimate, and their inclusion may introduce bias.

In practice, many case reports do not use standardization in the description of clinical symptoms and the course of hospitalization. For example, authors such as Albin et al. (2014) and Angus et al. (2013) omitted to mentioning the patient’s abdominal symptoms [34,36]. Such incomplete information makes it difficult to compare individual cases and weakens the power of the results and conclusions.

Furthermore, although we applied the Clavien–Dindo classification retrospectively in order to standardize the description of postoperative complications, this assessment was based solely on published clinical reports, which by their very nature may lead to an underestimation or overestimation of the severity of complications.

### 4.3. Limitations of the Review Process

Regarding the review methodology itself, there are also significant limitations that should be communicated. Firstly, we did not contact the authors of the analyzed publications, which would have allowed us to obtain supplementary data or correct any omissions in the reports. Separately, we made this choice deliberately because we were concerned that authors’ willingness to respond might be limited in older cases (e.g., pre-digital era) or that authors might be unavailable. Contacting the authors could favor more recent cases with better documented data, which would make older cases (with poorer documentation) less valuable for research and could introduce systematic bias.

Secondly, we did not conduct a formal assessment of the quality of reporting (e.g., in accordance with the CARE checklist for case descriptions). This was decided against because the subject matter of the work is rare and the literature is heterogeneous and limited.

Thirdly, the case search process was based on manual selection without full automation, which increases the risk of overlooking publications outside the main databases or those published in lesser-known languages or gray literature.

Last but not least, the inability to perform a quantitative meta-analysis and the lack of a control group limit the power of our statistical conclusions. The group of cases is small, and the heterogeneity and manner of describing the cases vary significantly, precluding robust comparative analysis regarding optimal management strategies. Therefore, our conclusions are mainly descriptive and generative in nature.

### 4.4. Practical Implications and Implications for Future Research

From a practical point of view, the results of our analysis suggest several key courses of action:

#### 4.4.1. Indications and Limitations of Conservative Treatment

The existing literature (although not in the context of trauma) has documented that conservative treatment may be considered in stable patients without signs of systemic infection or peritoneal symptoms (e.g., in perforated duodenal diverticula) [69]. In the post-traumatic context, however, we point out that, given the risk of progression to sepsis and the difficulty in controlling leakage, surgical treatment seems to be preferable. Conservative treatment is rarely used, in cases with a mild, oligosymptomatic course (without signs of peritonitis), in the elderly and in patients with multiple/chronic diseases [67].

#### 4.4.2. Justification for the Surgical Treatment

In post-traumatic cases, surgery allows not only for the removal of the source of perforation, but also for revision of the abdominal cavity and assessment of other internal organs, which are often damaged in multi-organ trauma. Due to the frequent coexistence of other abdominal injuries, the possibility of simultaneous action is important.

#### 4.4.3. Education of Clinicians

Emergency room physicians and surgeons should be specifically educated on recognizing the symptoms of D2 perforation in patients with seat belt injuries and abdominal injuries, even if the injury appears minor (e.g., no rupture of the skin, no external injuries). It is important to maintain high clinical vigilance—the symptoms are often non-specific (epigastric pain, tenderness, vomiting, nausea) and may mimic other conditions. In our analysis, drainage was used in 18/20 surgical cases, which appears to be a highly utilized element of therapy for the prevention of abscesses and biliary fistulas. Based on these observations, we suggest that routine drainage should be strongly considered in all such cases.

#### 4.4.4. Research Directions

Further analysis of the pathogenesis of perforation under conditions of increased intra-abdominal pressure is necessary—biomechanical models could help to determine the risk and mechanisms of diverticular rupture. Research on the relationship between the type of diverticulum (congenital vs. acquired), location and mechanism of injury (compression, deceleration, shear forces) is also indicated.

#### 4.4.5. Need for a Case Registry

We believe that the creation of a registry of causes related to duodenal diverticula in the context of injuries would be extremely valuable, as it would enable meta-analyses and prognostic analyses to be performed in the future. In particular, attention should be paid to: the paradoxically higher proportion of women in our data (despite the fact that men are more prone to injuries), the absence of cases in patients in their first and second decades of life, the very small number of patients under 40 years of age, and the large gap in histopathological data and lack of information on the nature of the diverticulum (congenital/acquired).

## 5. Conclusions

Perforations of the duodenal diverticulum following blunt trauma are a rare but potentially life-threatening complication, with a reported mortality rate of 4/21 in our analyzed cohort. In the reviewed literature, this complication predominantly affects elderly patients, most commonly after traffic accidents or falls from height. The aggregated data suggests that early diagnosis is crucial, as delays may lead to peritonitis, sepsis, and death. However, overall mortality and outcomes must be interpreted cautiously, as severe associated injuries (polytrauma) likely act as independent prognostic factors in these patients. High clinical vigilance is necessary in patients with abdominal trauma presenting with epigastric pain and free air on imaging. Early abdominal CT can facilitate timely recognition in suspected cases. Based on the available descriptive evidence, surgical intervention appears to be the most definitive management strategy, particularly in the presence of perforation and peritonitis.

## Figures and Tables

**Table 1:** Assessment of the risk of bias in case reports of duodenal diverticulum perforation following blunt trauma using the Joanna Briggs Institute (JBI) tool.

No.	Author (Year)	Reviewer 1										Reviewer 2										Results Risk of Bias		
		Demographics Clearly Described	History Timeline Presented	Clinical Condition on Presentation	Diagnostic Tests Results Described	Intervention Described	Post-Intervention Condition	Adverse Events Described	Takeaway Lessons	R1 RoB Score	R1 RoB Category	Demographics Clearly Described	History Timeline Presented	Clinical Condition on Presentation	Diagnostic Tests Results Described	Intervention Described	Post-Intervention Condition	Adverse Events Described	Takeaway Lessons	R2 RoB Score	R2 RoB Category	Consensus RoB Score	Consensus RoB Category	Agreement Rate (%)
**1**	***Brabrand (1960)*** [35]	**Y**	**Y**	**Y**	**Y**	**Y**	**U**	**Y**	**Y**	**7**	**L**	**Y**	**Y**	**Y**	**Y**	**Y**	**U**	**Y**	**Y**	**7**	**L**	**14**	**L**	**100**
**2**	***Graudins (1970)*** [27]	**Y**	**Y**	**Y**	**U**	**Y**	**U**	**Y**	**Y**	**6**	**L**	**Y**	**Y**	**Y**	**Y**	**Y**	**U**	**Y**	**Y**	**7**	**L**	**13**	**L**	**87.5**
**3**	***Ziman (1986)*** [23]	**Y**	**Y**	**Y**	**U**	**Y**	**Y**	**Y**	**Y**	**7**	**L**	**Y**	**Y**	**Y**	**U**	**Y**	**Y**	**Y**	**Y**	**7**	**L**	**14**	**L**	**100**
**4**	***Guglielmi (1993)*** [24]	**Y**	**Y**	**Y**	**Y**	**Y**	**Y**	**Y**	**Y**	**8**	**L**	**Y**	**Y**	**Y**	**Y**	**Y**	**Y**	**Y**	**Y**	**8**	**L**	**16**	**L**	**100**
**5**	***Souza (1996)*** [25]	**Y**	**Y**	**Y**	**U**	**Y**	**U**	**Y**	**Y**	**6**	**L**	**Y**	**Y**	**Y**	**U**	**Y**	**U**	**Y**	**Y**	**6**	**L**	**12**	**L**	**100**
**6**	***Poostizadeh (1997)*** [26]	**Y**	**Y**	**Y**	**Y**	**Y**	**Y**	**Y**	**Y**	**8**	**L**	**Y**	**Y**	**Y**	**Y**	**Y**	**Y**	**Y**	**Y**	**8**	**L**	**16**	**L**	**100**
**7**	***Atmani (2002)*** [37]	**Y**	**Y**	**Y**	**Y**	**Y**	**Y**	**Y**	**Y**	**8**	**L**	**Y**	**Y**	**Y**	**Y**	**Y**	**Y**	**Y**	**Y**	**8**	**L**	**16**	**L**	**100**
**8**	***Martinez (2006)*** [30]	**Y**	**U**	**Y**	**Y**	**Y**	**U**	**Y**	**Y**	**6**	**L**	**Y**	**U**	**Y**	**Y**	**Y**	**U**	**Y**	**Y**	**6**	**L**	**12**	**L**	**100**
**9**	***Fowler (2008)*** [31]	**Y**	**Y**	**Y**	**Y**	**Y**	**Y**	**Y**	**Y**	**8**	**L**	**Y**	**Y**	**Y**	**Y**	**Y**	**Y**	**Y**	**Y**	**8**	**L**	**16**	**L**	**100**
**10**	***Nazim (2009)*** [43]	**Y**	**Y**	**Y**	**Y**	**Y**	**Y**	**Y**	**Y**	**8**	**L**	**Y**	**Y**	**Y**	**Y**	**Y**	**Y**	**Y**	**Y**	**8**	**L**	**16**	**L**	**100**
**11**	***Metcalfe (2010)*** [40]	**Y**	**Y**	**Y**	**Y**	**Y**	**Y**	**Y**	**Y**	**8**	**L**	**Y**	**Y**	**Y**	**Y**	**Y**	**Y**	**Y**	**Y**	**8**	**L**	**16**	**L**	**100**
**12**	***Wedemeyer (2012)*** [28]	**Y**	**Y**	**Y**	**Y**	**Y**	**Y**	**Y**	**Y**	**8**	**L**	**Y**	**Y**	**Y**	**Y**	**Y**	**Y**	**Y**	**Y**	**8**	**L**	**16**	**L**	**100**
**13**	***Angus (2013)*** [36]	**Y**	**Y**	**U**	**Y**	**Y**	**Y**	**Y**	**Y**	**7**	**L**	**Y**	**Y**	**U**	**Y**	**Y**	**Y**	**Y**	**Y**	**7**	**L**	**14**	**L**	**100**
**14**	***Kim (2013)*** [32]	**Y**	**Y**	**Y**	**Y**	**Y**	**Y**	**Y**	**Y**	**8**	**L**	**Y**	**Y**	**Y**	**Y**	**Y**	**Y**	**Y**	**Y**	**8**	**L**	**16**	**L**	**100**
**15**	***Albin (2015)*** [34]	**Y**	**Y**	**U**	**Y**	**Y**	**Y**	**Y**	**Y**	**7**	**L**	**Y**	**Y**	**U**	**Y**	**Y**	**Y**	**Y**	**Y**	**7**	**L**	**14**	**L**	**100**
**16**	***Majerus (2015)*** [29]	**Y**	**Y**	**Y**	**Y**	**Y**	**Y**	**Y**	**Y**	**8**	**L**	**Y**	**Y**	**Y**	**Y**	**Y**	**Y**	**Y**	**Y**	**8**	**L**	**16**	**L**	**100**
**17**	***Palumbo (2020)*** [41]	**Y**	**Y**	**Y**	**Y**	**Y**	**Y**	**Y**	**Y**	**8**	**L**	**Y**	**Y**	**Y**	**Y**	**Y**	**Y**	**Y**	**Y**	**8**	**L**	**16**	**L**	**100**
**18**	***Shiraishi (2021)*** [39]	**Y**	**Y**	**Y**	**Y**	**Y**	**Y**	**Y**	**Y**	**8**	**L**	**Y**	**Y**	**Y**	**Y**	**Y**	**Y**	**Y**	**Y**	**8**	**L**	**16**	**L**	**100**
**19**	***Essofi (2023)*** [38]	**Y**	**Y**	**Y**	**Y**	**Y**	**Y**	**Y**	**Y**	**8**	**L**	**Y**	**Y**	**Y**	**Y**	**Y**	**Y**	**Y**	**Y**	**8**	**L**	**16**	**L**	**100**
**20**	***Staccini (2024)*** [42]	**Y**	**Y**	**Y**	**Y**	**Y**	**Y**	**Y**	**Y**	**8**	**L**	**Y**	**Y**	**Y**	**Y**	**Y**	**Y**	**Y**	**Y**	**8**	**L**	**16**	**L**	**100**
**21**	***Caires (2024)*** [33]	**Y**	**Y**	**Y**	**Y**	**Y**	**Y**	**Y**	**Y**	**8**	**L**	**Y**	**Y**	**Y**	**Y**	**Y**	**Y**	**Y**	**Y**	**8**	**L**	**16**	**L**	**100**

JBI—Joanna Briggs Institute. RoB—risk of bias. Y—Yes (meet the criteria). U—Unclear (Insufficient data). L—Low risk of bias. R1—Reviewer 1. R2—Reviewer 2.

**Table 2:** Demographic data, nature of injuries and clinical presentation of patients with blunt traumatic perforation of a duodenal diverticulum.

Variable		Value (n = 21)
**Demographics**	Age (years) *	62.9 (14.3)
	Sex (M:F)	8:13
**Mechanism of injury**	Traffic accident	10/21
	Fall from height	8/21
	Direct blow/kick	2/21
	Abdominal compression	1/21
**Location of diverticulum**	D2 segment	17/21
	D2 and D3 junction	2/21
	D4 segment	1/21
	Unknown/Not reported	1/21
**Type of diverticulum**	Congenital (true)	3/21
	Acquired (false)	7/21
	Unknown/Not reported	11/21
**Clinical presentation**	Abdominal pain	19/21
	Epigastric tenderness	16/21
	Peritoneal signs	10/21
	Nausea/vomiting	5/21

* Values are n unless otherwise indicated. Values are mean (SD) (median 64, range 33–84).

**Table 3:** Patient demographics and clinical presentation.

No.	Study (Year)	Age/Sex	Mechanism of Injury	Diverticulum (Loc./Type)	Clinical Symptoms	Delayed Peritoneal Signs	Dx Method
**1**	Brabrand (1960) [35]	54/F	Fall against bed	D2/Ac	AP, NV, ET, PS, F	No	Lap.
**2**	Graudins (1970) [27]	70/F	Slip and fall	D2/Co	AP, NV, ET, PS	No	Lap.
**3**	Ziman (1986) [23]	53/M	Fall from scaffolding (6 m)	D2/NR	AP, ET, PS	Yes (4 h)	Lap.
**4**	Guglielmi (1993) [24]	33/F	Fall from height (3 m)	D2/NR	AP, ET, PS	No	CT
**5**	Souza (1996) [25]	49/M	Traffic accident	D2/Co	AP, ET	No	Lap.
**6**	Poostizadeh (1997) [26]	72/F	Traffic accident	D2/Ac	AP, ET	No	CT
**7**	Atmani (2002) [37]	83/F	Traffic accident	D2/NR	AP, ET	No	CT
**8**	Martinez (2006) [30]	73/M	Fall downstairs	D2/NR	AP, ET, PS	No	CT
**9**	Fowler (2008) [31]	45/F	Fall from bucket	D2/NR	AP, NV, ET, PS, F	Yes (1 d)	CT
**10**	Nazim (2009) [43]	84/F	Traffic accident	D2/NR	AP	No	CT
**11**	Metcalfe (2010) [40]	58/M	Fall from ladder	D2/D3/NR	AP, ET, PS	No	CT
**12**	Wedemeyer (2012) [28]	79/F	Fall (crush against railing)	D2/NR	AP, ET, PS	No	CT
**13**	Angus (2013) [36]	64/F	Traffic accident	D2/NR	NR	No	CT
**14**	Kim (2013) [32]	61/M	Fall downstairs	D2/D3/NR	AP	No	CT
**15**	Albin (2015) [34]	65/F	Traffic accident	D2/Ac	NR	No	CT
**16**	Majerus (2015) [29]	65/F	Traffic accident	D2/Ac	AP, NV, ET	No	CT
**17**	Palumbo (2020) [41]	82/M	Traffic accident	D4/Ac	AP, NV, ET, PS	No	CT
**18**	Shiraishi (2021) [39]	67/F	Kick to abdomen	D2/Ac	AP, ET	No	CT
**19**	Essofi (2023) [38]	63/M	Traffic accident	NR/NR	AP	Yes (2 d)	CT
**20**	Staccini (2024) [42]	36/M	Kick to abdomen	D2/Co	AP, ET, PS	No	CT
**21**	Caires (2024) [33]	64/F	Traffic accident	D2/NR	AP, ET	No	CT

F—female. M—male. D2—descending part of the duodenum. D3—horizontal part of the duodenum. D4—ascending part of the duodenum. Ac—acquired. Co—congenital. AP—abdominal pain. NV—nausea/vomiting. ET—epigastric tenderness. PS—peritoneal symptoms. F (in symptoms)—fever. CT—computed tomography. Lap.—laparotomy. NR—not reported (no information).

**Table 4:** Clinical management and outcomes.

No.	Study (Year)	Associated Injuries	Tx Method (Drainage)	Time to Dx/Time to Tx	Complications (Clavien–Dindo)	LOS (Days)
***1***	*Brabrand (1960)* [35]	*None*	*Surg. (L)/Yes*	*4 d/4 d*	*IIIa*	*49*
***2***	*Graudins (1970)* [27]	*None*	*Surg. (L)/Yes*	*5 h/5 h*	*V*	*4*
***3***	*Ziman (1986)* [23]	*None*	*Surg. (L)/Yes*	*7 h/7 h*	*II*	*25*
***4***	*Guglielmi (1993)* [24]	*None*	*Surg. (L)/Yes*	*FH/FH*	*II*	*21*
***5***	*Souza (1996)* [25]	*Yes (Orthopedic)*	*Surg. (L)/Yes*	*FH/ES*	*V*	*68*
***6***	*Poostizadeh (1997)* [26]	*None*	*Surg. (L)/Yes*	*FH/FH*	*V*	*3*
***7***	*Atmani (2002)* [37]	*None*	*Surg. (L)/Yes*	*FH/FH*	*None*	*21*
***8***	*Martinez (2006)* [30]	*None*	*Surg. (L)/Yes*	*FH/FH*	*None*	*NR*
***9***	*Fowler (2008)* [31]	*None*	*Surg. (SL)/Yes*	*1 d/1 d*	*None*	*4*
***10***	*Nazim (2009)* [43]	*None*	*Surg. (SL)/Yes*	*4 h/ES*	*IVa*	*21*
***11***	*Metcalfe (2010)* [40]	*None*	*Surg. (L)/Yes*	*ES/ES*	*II*	*11*
***12***	*Wedemeyer (2012)* [28]	*None*	*Surg. (SL)/Yes*	*FH/ES*	*None*	*12*
***13***	*Angus (2013)* [36]	*Severe (Thoracic, Abdominal, Orthopedic)*	*Surg. (SL)/Yes*	*FH/ES*	*IIIa*	*7*
***14***	*Kim (2013)* [32]	*Yes (Abdominal)*	*Surg. (L)/Yes*	*3 h/ES*	*V*	*10*
***15***	*Albin (2015)* [34]	*Severe (Thoracic, Abdominal, Orthopedic)*	*Surg. (L)/Yes*	*FH/ES*	*None*	*5*
***16***	*Majerus (2015)* [29]	*None*	*Surg. (SL)/Yes*	*FH/FH*	*II*	*12*
***17***	*Palumbo (2020)* [41]	*Yes (Neurological, Cervical)*	*Surg. (SL)/Yes*	*FH/FH*	*None*	*NR*
***18***	*Shiraishi (2021)* [39]	*None*	*Surg. (SL)/No*	*6 h/ES*	*None*	*21*
***19***	*Essofi (2023)* [38]	*Yes (Orthopedic)*	*Conservative*	*NR/Cons.*	*None*	*NR*
***20***	*Staccini (2024)* [42]	*None*	*Surg. (SL)/Yes*	*2 h/4 h*	*None*	*10*
***21***	*Caires (2024)* [33]	*Severe (Thoracic, Cervical, Orthopedic)*	*Surg. (SL)/No*	*FH/ES*	*IVa*	*7*

Dx—diagnosis. Tx—treatment. Surg. (L)—surgical treatment (laparotomy). Surg. (SL)—surgical treatment (stapled laparotomy). Cons.—conservative treatment. FH—few hours (imprecise timeframe retained exactly as reported). ES—emergency surgery (imprecise timeframe retained exactly as reported). LOS—length of stay (days). NR—not reported (no information). Clavien–Dindo—retrospective grading of complications (highest grade developed; Grade V indicates death).

**Table 5:** Diagnostic methods, management and clinical outcomes.

Category	Sub-Category	Modality/Parameter	Value (n = 21)
**Imaging performed**		Computed tomography (CT)	17/21
		Ultrasonography	5/21
		Radiography (X-ray)	5/21
**Management strategy**	Surgical intervention	Total underwent surgery	20/21
		Manual diverticulectomy	11/21
		Stapled diverticulectomy	9/21
		Abdominal drainage applied	18/21
	Conservative care	Total treated conservatively	1/21
**Clinical outcomes**	Hospitalization	Length of stay (days) *	17.3 (16.8)
	Morbidity	Total complications	13/21
		Minor complications †	5/21
		Severe complications ‡	8/21
	Mortality	Total deaths	4/21
	Follow-up	Time (months)	1–12 (average 5.4)

* Values are n unless otherwise indicated. Values are mean (SD) (median 11.5, range 3–68). † Includes wound infection, periduodenal abscess, pleural effusion, and atelectasis. ‡ Includes biliary fistula, sepsis, multiple organ failure, pneumonia, intestinal obstruction, and pulmonary embolism.

## Data Availability

All data generated or analyzed during this study are included in this published article. The detailed PRISMA checklist (Supplementary Table S1) and literature search strategy (Supplementary File S1) are available in the online Supplementary Materials.

## Supplementary Materials

The following supporting information can be downloaded at: https://www.mdpi.com/article/10.3390/jcm15114390/s1, Table S1: PRISMA checklist; File S1: Literature search strategy. Reference [70] is cited in Supplementary Materials.

## Abbreviations

The following abbreviations are used in this manuscript:

CARE	Case Report Guidelines
CT	Computed Tomography
D1, D2, D3, D4	parts of the duodenum:
D1	(ampulla)
D2	(Descending part)
D3	(Horizontal part)
D4	(Ascending part)
DD	Duodenal Diverticulum
eFAST/FAST	(Extended) Focused Assessment with Sonography in Trauma
ERCP	Endoscopic Retrograde Cholangiopancreatography
JBI	Joanna Briggs Institute
MRI	magnetic resonance imaging
PRISMA	Preferred Reporting Items for Systematic Reviews and Meta-Analyses
PROSPERO	International prospective register of systematic reviews
RoB	Risk of Bias
SD	standard deviation
USG	Ultrasonography (ultrasound)
X-ray	Radiography
