# Supplementary material for: Diagnostic Challenges and Management of Blunt Traumatic Duodenal Diverticulum Perforation: A Systematic Review
Source: J Clin Med. 2026 Jun 5;15(11):4390. doi: 10.3390/jcm15114390 (PMC13258038; doi:10.3390/jcm15114390)
Supplement: Supplementary file 1 [file jcm-15-04390-s001.zip › PROSPERO.pdf]

Diagnostic Challenges and Management of Blunt Traumatic Duodenal Diverticulum Perforation: A Systematic Review

Karol Kłosiński, Maciej Rybicki, Bartłomiej Białas, Zbigniew Pasieka, Bartosz Czyżewski, Piotr Arkuszewski

Citation
Karol Kłosiński, Maciej Rybicki, Bartłomiej Białas, Zbigniew Pasieka, Bartosz Czyżewski, Piotr Arkuszewski. Diagnostic Challenges and Management of Blunt Traumatic Duodenal Diverticulum Perforation: A Systematic Review. Not yet published.

PREVIEW

REVIEW TITLE AND BASIC DETAILS

Review title
Diagnostic Challenges and Management of Blunt Traumatic Duodenal Diverticulum Perforation: A Systematic Review

Condition or domain being studied
Diverticulectomy Of Duodenum; Abdominal surgery; Trauma; Intestinal Perforation; Duodenum disorder; Laparotomy; Disorder Of Digestive System; Computed tomography; Drainage Procedure; Morbidity Index; Postoperative Complication

Rationale for the review
Perforation of the duodenal diverticulum as a result of blunt abdominal trauma remains one of the rarest and most insidious pathologies in trauma surgery, with a high mortality rate and extremely difficult diagnosis. This paper fills a significant gap in the global literature, as it's the first and only systematic review covering all available cases published between 1960 and 2025, allowing us to create the most comprehensive synthesis of clinical knowledge in this field.

Analysis was performed in accordance with PRISMA 2020 guidelines, and the quality of each case was assessed using the JBI Critical Appraisal Checklist, resulting in a uniformly low risk of systematic error, which significantly strengthens the reliability of our conclusions. In this paper, we also present our own biomechanical model explaining the mechanisms leading to perforation, based on the analysis of shear forces and the closed-loop phenomenon, illustrated with original diagrams prepared specifically for this review. We were the first to attempt a systematic analysis of clinical symptoms, which allows for a better understanding of the causes of diagnostic delays. Our results also indicate the very high effectiveness of computed tomography and the particular susceptibility of the descending duodenum (D2) to injury, which is of direct relevance to radiological and surgical practice.

Review objectives
The overall objective of this systematic review is to collect, analyze, and summarize all published cases of duodenal diverticulum perforation associated with blunt abdominal trauma. The review aims to characterize the clinical profile and treatment methods used in this rare condition by answering the following research questions:

1. Demographic characteristics and mechanisms of injury: what are the typical characteristics of patients and the mechanisms (e.g., compression, deceleration) leading to perforation?

2. Clinical presentation and diagnosis: what are the predominant symptoms and what is the diagnostic value of imaging studies (CT, ultrasound, X-ray)?

3. Treatment outcomes: what are the therapeutic effects, complication rates, and mortality rates depending on the strategy used (surgical vs. conservative)?

Keywords
Blunt abdominal trauma; Duodenal Diverticulum Perforation; Gastrointestinal surgery; Computed tomography; Systematic review

Country
Poland

ELIGIBILITY CRITERIA

Population

Included
retrospective papers , case reports and case series , duodenal diverticulum rupture as a result of blunt trauma , duodenal diverticulum rupture had been confirmed surgically or by using imaging tests, publications issued between 1960 and 2025 , articles with an available abstract.

Excluded
Stab wounds , iatrogenic injuries , injuries of unclear etiology

Intervention(s) or exposure(s)

Included
Excision Of Diverticulum Of Small Intestine; Excision Of Diverticulum Of Ampulla Of Vater; Diverticulectomy Of Duodenum; Operation On Duodenum; Abdominal surgery; Abdominal Drain

blunt abdominal trauma leading to confirmed perforation of the duodenal diverticulum

any surgical management (for example diverticulectomy, stapled diverticulectomy, drainage) as well as conservative treatment

Excluded
Penetrating trauma (e.g., stab wounds), Iatrogenic injuries (e.g., post-ERCP perforation), Injuries of unclear etiology

Comparator(s) or control(s)
This review does not have any comparators

Study design
Only nonrandomized study types will be included.

Included
Case studies, case series

Context
Inclusion: retrospective papers , case reports and case series , duodenal diverticulum rupture as a result of blunt trauma , duodenal diverticulum rupture had been confirmed surgically or by using imaging tests, publications issued between 1960 and 2025 , articles with an available abstract.

Exclusion: Stab wounds , iatrogenic injuries , injuries of unclear etiology

SIMILAR REVIEWS

Check for similar records already in PROSPERO
This is the first systematic review of this kind in the world.

TIMELINE OF THE REVIEW

Date of first submission to PROSPERO
This record has not been submitted.

Review timeline
Start date: 1 September 2025. End date: 12 February 2026.

Date of registration in PROSPERO
This record has not been published.

AVAILABILITY OF FULL PROTOCOL

Availability of full protocol
A full protocol has been written but is not available because:

There are no plans to publish the full search protocol due to the advanced nature of the work and the plan to publish the results of the work with an extensive description of the search in the near future.

SEARCHING AND SCREENING

Search for unpublished studies
Only published studies will be sought.

Main bibliographic databases that will be searched
The main databases to be searched are *Embase - Embase via Ovid*, *MEDLINE*, *PubMed* and *Scopus*.

Other important or specialist databases that will be searched
Web of Science, Cochrane Library Databases (Google scholar)

Search language restrictions
There are no language restrictions.

Search date restrictions
Databases will be searched for articles published from 1 January 1960 and before by 31 December 2025.

Other methods of identifying studies
Other studies will be identified by: *looking through all the articles that cite the papers included in the review ("snowballing" or forward citation searching)* and *reference list checking (backward citation searching)*.

Additional information about identifying studies
In articles that meet the inclusion criteria, the bibliography will be searched for other articles that may be relevant to the research topic (snowballing).

Due to the broad time frame and to ensure fairness, there are no plans to contact authors for additional information (this could favour newer publications.

Link to search strategy
A full search strategy has been uploaded to PROSPERO. The PDF may be accessed through this link https://www.crd.york.ac.uk/PROSPEROFILES/9460dda1d2c62e1fd4bda6022a2d4f4f.pdf.

Selection process
Studies will be screened independently by at least two people (or person/machine combination) with a process to resolve differences.

Other relevant information about searching and screening
The search was not restricted to articles in English.

Duplicate data were excluded, only the original data from the author describing the case were included.

No contact with authors was planned to supplement missing data; all information was obtained directly from publications.

DATA COLLECTION PROCESS

Data extraction from published articles and reports
Data will be extracted independently by at least two people (or person/machine combination) with a process to resolve differences.

Authors will not be contacted for further information.

Study risk of bias or quality assessment
Risk of bias will be assessed using:

Joanna Briggs Institute

Data will be assessed independently by at least two people (or person/machine combination) with a process to resolve differences.

Additional information will **not** be sought from study investigators if required information is unclear or unavailable in the study publications/reports.

Reporting bias assessment
Risk of bias due to missing results will not be assessed

Certainty assessment

Certainty of findings will not be assessed

OUTCOMES TO BE ANALYSED

Main outcomes

Length of hospitalization, patient outcome, summary of symptoms, characteristics of diverticula, treatment methods, treatment outcomes

Additional outcomes

Location of diverticulum, size, nature (congenital/acquired), clinical symptoms (abdominal pain, vomiting, epigastric tenderness, peritoneal symptoms, elevated temperature, others), diagnostic method, histopathological result (if performed) surgical treatment method, presence of drainage, complication

PLANNED DATA SYNTHESIS

Strategy for data synthesis

Due to the heterogeneous and probably small group of cases, no extensive statistical analysis is planned. It is planned to calculate simple statistical variables such as: means, deviations, medians, etc.

CURRENT REVIEW STAGE

Stage of the review at this submission

Review stage	Started	Completed
Pilot work	✓	✓
Formal searching/study identification	✓	✓
Screening search results against inclusion criteria	✓	✓
Data extraction or receipt of IPD		
Risk of bias/quality assessment		
Data synthesis		

Review status

The review is currently planned or ongoing.

Publication of review results

Results of the review will be published in English and Polish.

REVIEW AFFILIATION, FUNDING AND PEER REVIEW

Review team members

Dr Karol Kłosiński. ORCID: 0000-0003-0962-228X. Medical University of Lodz. Poland.

No conflict of interest declared.

Mr Maciej Rybicki. ORCID: 0009-0000-5584-1075. Plastic, Reconstructive and Aesthetic Surgery Clinic, Institute of Surgery, Medical University of Lodz, Lodz, Poland; Department of Biomedicine and Experimental Surgery, Faculty of Medicine, Medical University of Lodz, Narutowicza 60, 90-136 Lodz, Poland. Poland.

No conflict of interest declared.

Mr Bartłomiej Białas. ORCID: 0009-0007-6492-9096. Department of Biomedicine and Experimental Surgery, Faculty of Medicine, Medical University of Lodz, Narutowicza 60, 90-136 Lodz, Poland. Poland.

No conflict of interest declared.

Associate Professor Zbigniew Pasieka. ORCID: 0000-0001-8931-0213. Department of Biomedicine and Experimental Surgery, Faculty of Medicine, Medical University of Lodz, Narutowicza 60, 90-136 Lodz, Poland; Department of Endocrine, General and Oncological Surgery, The Nicolaus Copernicus Provincial Multispecialty Center for Oncology and Traumatology in Lodz. Poland.

No conflict of interest declared.

Mr Bartosz Czyżewski. ORCID: 0009-0005-1665-8573. Department of Biomedicine and Experimental Surgery, Faculty of Medicine, Medical University of Lodz, Narutowicza 60, 90-136 Lodz, Poland. Poland.

No conflict of interest declared.

Dr Piotr Arkuszewski (review guarantor and contact) ORCID: 0000-0002-7151-7547. Department of Biomedicine and Experimental Surgery, Faculty of Medicine, Medical University of Lodz, Narutowicza 60, 90-136 Lodz, Poland. Poland.

No conflict of interest declared.

Named contact

Dr Piotr Arkuszewski (piotr.tomasz.arkuszewski@umed.lodz.pl). ORCID: 0000-0002-7151-7547. Department of Biomedicine and Experimental Surgery, Faculty of Medicine, Medical University of Lodz, Narutowicza 60, 90-136 Lodz, Poland. Poland.

Review affiliation

Medical University of Lodz

Funding source

Review has no specific/external funding but is supported by guarantor/review team (non-commercial) institutions.

Additional information about funding

This study received no external funding. The research will be conducted as part of academic activity within the Medical University of Lodz

Peer review

There has been no peer review of this planned review.

ADDITIONAL INFORMATION

Additional information

The following systematic review fills an important research gap. Due to the small number of cases (+- 20), no meta-analysis or formal analysis of heterogeneity or subgroups will be performed, but the synthesis of data allowed for the creation of a comprehensive clinical picture.

Review conflict of interest

Declared individual interests are recorded under team member details.. No additional interests are recorded for this review.

Medical Subject Headings

Conservative Treatment; Deceleration; Demography; Diverticulum; Drainage; Humans; Tomography, X-Ray Computed; Treatment Outcome; X-Rays

PROSPERO version history

No preview available

Disclaimer

The content of this record displays the information provided by the review team. PROSPERO does not peer review registration records or endorse their content.

PROSPERO accepts and posts the information provided in good faith; responsibility for record content rests with the review team. The guarantor for this record has affirmed that the information provided is truthful and that they understand that deliberate provision of inaccurate information may be construed as scientific misconduct.

PROSPERO does not accept any liability for the content provided in this record or for its use. Readers use the information provided in this record at their own risk.

Any enquiries about the record should be referred to the named review contact
