# Supplementary material for: Diagnostic Challenges and Management of Blunt Traumatic Duodenal Diverticulum Perforation: A Systematic Review
Source: J Clin Med. 2026 Jun 5;15(11):4390. doi: 10.3390/jcm15114390 (PMC13258038; doi:10.3390/jcm15114390)
Supplement: Supplementary file 1 [file jcm-15-04390-s001.zip › Search strategy SR DD.pdf]

PubMed/MEDLINE:

("Duodenal Diverticulum"[Mesh] OR "duodenal diverticulum*" OR "diverticulum of the duodenum" OR "duodenal diverticuli")

AND

("Trauma"[Mesh] OR trauma OR injury OR perforation OR rupture OR "blunt trauma" OR "penetrating trauma" OR "iatrogenic injury" OR "traumatic perforation" OR "traumatic rupture")

SCOPUS:

(TITLE-ABS-KEY("duodenal diverticulum" OR "duodenal diverticula" OR "diverticulum of the duodenum"))

AND

(TITLE-ABS-KEY("trauma" OR "injury" OR "perforation" OR "rupture" OR "blunt trauma" OR "penetrating trauma" OR "iatrogenic injury" OR "traumatic perforation" OR "traumatic rupture"))

Web Of Science:

TS=("duodenal diverticulum" OR "duodenal diverticula") AND TS=(trauma OR injury OR traumatic OR "blunt trauma" OR perforation OR perforated OR crush OR compression OR rupture OR laceration)

Embase (OVID):

('duodenal diverticulum'/exp OR 'duodenal diverticulum':ti,ab)

AND

('duodenal injury'/exp OR trauma*:ti,ab OR injur*:ti,ab OR perforat*:ti,ab OR ruptur*:ti,ab)

Cochrane Library:

("duodenal diverticulum" OR "duodenal diverticula") AND (trauma OR "blunt trauma" OR perforation OR perforated OR rupture) NOT poster NOT protocol NOT editorial NOT letter.

Google scholar:

"duodenal diverticulum" AND (trauma OR injury OR perforation OR rupture OR "blunt trauma" OR "penetrating trauma" OR "iatrogenic injury" OR "traumatic perforation" OR "traumatic rupture")
